# Colony-Level Efficacy of *Mentha piperita*, *Thymus vulgaris* and *Eucalyptus globulus* Essential Oil Nanoemulsions Against *Varroa destructor*

**DOI:** 10.1007/s10493-026-01141-y

**Published:** 2026-05-05

**Authors:** Mustafa Güneşdoğdu

**Affiliations:** Department of Animal Production and Technologies, Faculty of Applied Sciences , Muş Alparslan University , Muş, Turkey

**Keywords:** *Apis mellifera*, Natural acaricide, Pesticide formulation, Nanotechnology, *Varroa* mortality

## Abstract

Honey bees (*Apis mellifera* L.) are essential for ecosystem stability and agricultural production; however, one of the most destructive threats to colonies is the mite *Varroa destructor*. Although conventional chemical acaricides can provide short-term control, resistance development and residue concerns remain major challenges. Plant-derived essential oils have therefore attracted attention as alternative control agents. In this study, the field efficacy of nanoemulsions (NEms) prepared from peppermint (*Mentha piperita*; P-NEms), thyme (*Thymus vulgaris*; T-NEms), and eucalyptus (*Eucalyptus globulus*; E-NEms) oils against *Varroa* mites was evaluated at three doses (50, 100, and 200 ppm), and compared with a positive control (amitraz) and a negative control (no treatment). All tested nanoemulsion formulations significantly reduced *Varroa* mite density, with the highest efficacy (percentage reduction relative to initial mite counts) observed at the 200 ppm dose. The T-NEms200 treatment exhibited the strongest acaricidal activity (75.3%). Peppermint and eucalyptus NEms also achieved high efficacy. The positive control, amitraz, showed 53.5% efficacy and was less effective than the higher NEms doses, while mite loads increased in untreated colonies. The nanoscale droplet structure of the formulations may have contributed to enhanced contact between active compounds and mites. Overall, these findings demonstrate that essential oil-based nanoemulsions reduced *Varroa* mite populations in a concentration-dependent manner at the colony level. However, the present study focused on efficacy, and further research is required to evaluate potential effects on bee health, brood development, and residue levels in hive products before practical recommendations can be made.

**Graphical abstract:**

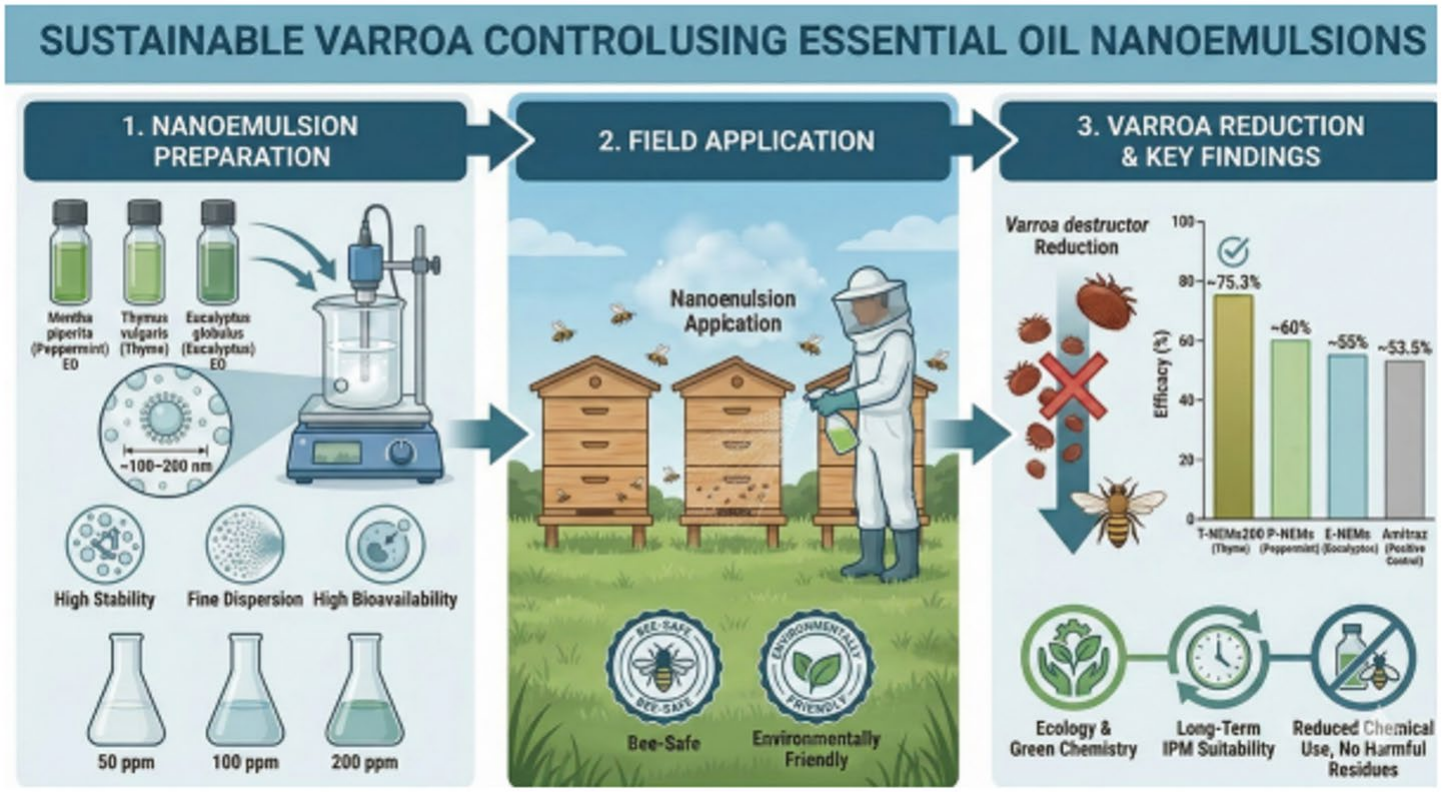

**Supplementary Information:**

The online version contains supplementary material available at 10.1007/s10493-026-01141-y.

## Introduction

Honey bees and other pollinators play a crucial role in global food production and nutritional security (Rajagopalan et al. 2024). In recent years, however, honey bee colonies have faced serious threats (Sönmez Oskay et al. 2025). Foremost among these is the ectoparasite *Varroa destructor* (formerly *V. jacobsoni* Oudemans*)* (Anderson and Truemann, 2000). This parasite feeds on bee larvae and adults, and and feeds on both fat bodies and hemolymph (Han et al. 2024). The *Varroa* mite transmits various viruses to honey bees, including deformed wing virus (DWV), acute bee paralysis virus (ABPV, and Kashmir bee virus (KBV) (Lester et al. 2022), resulting in poor fitness and impaired immune system development, which weakens colonies and leads to mortality (Rosenkranz et al. 2010). This has become a major problem, threatening the health of both individual colonies and the overall bee population.

Chemical acaricides have traditionally been preferred for controlling *Varroa* mites (Jack and Ellis 2021). However, long-term use of these chemicals can lead to resistance in mites and may cause adverse effects such as negative impacts on bee health and residue accumulation in hive products (Güneşdoğdu et al. 2022). Consequently, interest in natural control methods for *Varroa* mites has increased in the beekeeping sector in recent years. Essential oils (EOs) prove to be promising candidates due to their good efficacy and good environmental biodegradability (Bava et al. 2024). Some studies have indicated that essential oils can exhibit acaricidal activity comparable to synthetic chemical treatment (Khajehali et al. 2023; Narciso et al. 2024), whereas others report only moderate efficacy that falls short of the requirements for commercial product development (Bava et al. 2024). In addition, several authors have reported that some essential oils not only shows promising efficacy against *Varroa* mites (Alsaadi et al. 2024) but may also exert repellent effects even when they are not directly lethal (Alahyane et al. 2022). While current findings indicate the potential of essential oils, they also show that both the risks they may pose to honey bees (Da Silva et al. 2020) and their potential benefits (Conti et al. 2020) should be carefully evaluated. Therefore, more comprehensive studies demonstrating the direct acaricidal effects of essential oils as biopesticides are still needed (Gostin and Popescu 2023). Essential oils are natural compounds derived from plants (roots, leaves, flowers, and seeds) that possess antimicrobial, antifungal, and insecticidal properties (Reyes-Jurado et al. 2015). Essential oils are considered an attractive alternative in pest management due to their natural origin, easy availability, and intrinsic insecticidal properties derived from the plant defense system. Their lower toxicity to humans and non-target organisms compared to synthetic insecticides makes them a suitable option for integrated pest management (IPM) and organic production approaches (Isman 2023).

Unlike conventional chemical pesticides, essential oils are preferred as they are generally less susceptible to the rapid emergence of resistance, a characteristic attributed to the synergistic effects of their diverse bioactive constituents (Isman 2020). The most commonly used essential oils as biopesticides with insecticidal or acaricidal activity are citronella oil, lemongrass oil, clove oil, peppermint oil, cinnamon oil, rosemary oil, sweet orange oil, eucalyptus oil, and thyme oil (Isman and Machial 2006). Thymol is a volatile monoterpenoid and a natural component of thyme (*Thymus vulgaris*). It is one of the most widely used essential oil–based compounds for controlling *Varroa* mites (Price and Lummis 2022) and is included in several nationally registered veterinary medicinal products, such as Apiguard^®^, Thymovar^®^, and ApiLife Var^®^. Thymol and menthol have inhibitory effects on the growth and reproduction of insect and mite pests (Kraus et al. 1994). Although thymol is widely used in *Varroa* control, its application may result in detectable residues in honey (Sánchez et al. 2021) and beeswax (Kast et al. 2022). However, under recommended use conditions, these residues have not been reported to pose hygienic risks to bees or humans (Adamczyk et al. 2005). Peppermint oil also has a lethal effect on mites due to its high menthol (55%) content (Da Silva et al. 2020). Oil obtained from the leaves of eucalyptus trees, which include more than 800 species (Polito et al. 2023), targets mites due to the 1,8-cineole compound it contains and has properties that support the immune system of bees (Atmani-Merabet et al. 2020). However, the short persistence of essential oils due to rapid evaporation and degradation from environmental conditions can require repeated applications and increase costs. Therefore, developing new formulations and application techniques to increase the effectiveness of essential oils represents a significant opportunity in pest control (Campolo et al. 2017).

Nanotechnology has emerged as a key technological field in the 21 st century. Nanoparticles and nanoformulations can be developed from both organic and inorganic substances (Bhushan et al. 2014). The development of nano-based pesticides for agriculture is an active area of research (Deka et al. 2021). Due to their small size and large surface area, nanoproducts can exhibit enhanced biological activity (Yan et al. 2022). One of the frequently cited advantages of essential oils is their generally rapid environmental degradation and lower persistence compared to many synthetic pesticides. However, volatility and stability issues can limit their effectiveness. Nanoemulsion (NEms) technology has been developed to overcome these challenges. NEms are colloidal systems composed of nanometer-sized droplets that combine two immiscible phases, such as oil and water (Elzayat et al. 2021). A key advantage of NEms is their ability to enhance the bioavailability of essential oils and optimize their effects on target organisms through controlled release mechanisms (Kumar et al. 2025). To date, only a limited number of studies have investigated the effectiveness of essential oil–based nanoemulsions against *Varroa* mites (El Roby et al. 2023; Gamal Eldin et al. 2024). The use of essential oils represents a safer and environmentally sustainable alternative, as they do not pose a risk of chemical residues in honey bee products. In this context, essential oils and NEms are increasingly recognized as biotechnological tools supporting both bee health and ecological sustainability. Nano-formulations may offer advantages over conventional control methods; however, studies specifically targeting *V. destructor* remain limited, highlighting the need for further applied research under diverse regional and colony conditions (Farina et al. 2024). Recently, Gamal Eldin et al. (2024) evaluated an indirect application approach using a single active compound (thymol NEms) incorporated into candy sugar, which restricts the range of application strategies reported in the literature. In contrast, the present study developed three essential oil-based NEms (thyme, peppermint, and eucalyptus) and applied them directly to bee-covered frames via spraying. This direct application strategy represents an important advancement in formulation diversity and delivery approach, enabling faster, more uniform, and potentially more effective exposure of colonies to bioactive compounds.

Therefore, the aim of this study was to evaluate the colony-level efficacy of nanoemulsions formulated with *Mentha piperita*, *Thymus vulgaris*, and *Eucalyptus globulus* essential oils against *Varroa destructor* under field conditions. Specifically, the study assessed concentration-dependent effects on mite infestation levels and compared the performance of these formulations with a conventional acaricide treatment.

## Materials and methods

### Oil materials and compounds

Thyme, peppermint, and eucalyptus essential oils were purchased from a local herbal products market (Bitki Evim, Izmir, Türkiye). The oils were stored in a refrigerator at + 4 °C until further analysis and emulsification. The chemical composition of the essential oils was determined following the methodology outlined by Ligor et al. (2014), with minor modifications. A gas chromatography–mass spectrometry (GC-MS) (PerkinElmer Inc., Waltham, Massachusetts, USA), was used for this analysis. Separation was performed using a non-polar fused silica capillary column (SGE BPX5, 60 m × 0.25 mm ID, 0.25 μm film thickness, USA). The oven temperature was initially set at 60 °C and maintained for 10 min, then increased at a rate of 4 °C/min to 250 °C and held for an additional 10 min. The injector temperature was maintained at 220 °C. Helium was used as the carrier gas at a constant flow rate of 1.5 mL/min. 1 µL sample of each essential oil, previously diluted in hexane (1:100, v/v), was injected in splitless mode. Mass spectrometric detection was performed using electron ionization (EI) at 70 eV. The ion source and interface temperatures were set at 200 °C and 250 °C, respectively. Mass spectra were recorded in the m/z 35–425 range. Volatile components were identified by comparing the obtained spectra with those in the NISTMS and WILEYMS 9 spectral libraries (National Institute of Standards and Technology, USA).

### Preparation of nanoemulsions

Essential oil-based nanoemulsions were prepared using a modified version of the method described by Joe et al. (2012). The nanoemulsion formulations consisted of essential oil (thyme, peppermint, or eucalyptus) as the oil phase, Tween 80 as the primary surfactant, ethanol as the co-surfactant, and double-distilled water as the aqueous phase. The ingredients and their respective amounts for the different NEms (O/W type) doses are shown in Table 1. According to the procedure, all ingredients except water were first mixed and heated at 86 °C for 1 h to promote ethanol evaporation. A similar approach has been described in nanoemulsion preparation protocols, where ethanol evaporation was assumed at temperatures above its boiling point (78.4 °C) (Özoğul et al. 2017). However, residual ethanol levels were not analytically quantified in the present study. The remaining mixture was then diluted with double-distilled water according to the proportions indicated in Table 1. Before ultrasonic homogenization, the mixture was pre-mixed using a mechanical homogenizer (IKA T25 digital ULTRA TURRAX) at 500 rpm for 15 min. The final emulsion was then processed for 15 min at 72% amplitude using an ultrasonic homogenizer (Optic Ivymen System CY-500, Barcelona, Spain). The device operated at 500 W and 20 kHz. The sonotrode used was 5.6 mm in diameter and 60 mm in length. During sonication, the emulsion temperature was maintained at 15 °C using an ice bath. The final volume of each nanoemulsion was 500 ml. Tween 80 was selected as a non-ionic surfactant due to its widespread use in nanoemulsion systems and its reported compatibility in honey bee-related applications, with no significant adverse effects observed under laboratory conditions (Wang et al. 2025). Ethanol was used as a co-surfactant in the nanoemulsion system. Co-surfactants such as Transcutol P, glycerol, ethylene glycol, ethanol, propanol, isopropanol, n-butanol, PEG 400, carbitol, and propylene glycol are widely used in nanoemulsion formulations to promote emulsification at low surfactant concentrations (Kreilgaard et al. 2000). Alcohol derivatives help reduce interfacial tension, improve interfacial fluidity, and increase the mobility of hydrocarbon chains, facilitating better penetration of the oil phase into the interfacial region (Tenjarla 1999). Additionally, alcohol distributes between the two phases and increases their miscibility (Chime et al. 2014).

**Table 1:** Composition of nanoemulsion formulations at different essential oil concentrations

NEms Dose	Essential Oil Level	Surfactant (Tween 80)	Ethanol	Water	Total Volume
50 ppm	0.25 ml	5 ml	15 ml	479.75 ml	500 ml
100 ppm	0.5 ml	5 ml	15 ml	479.50 ml	
200 ppm	1 ml	5 ml	15 ml	479.00 ml	

### Physical properties of emulsions

The average droplet size of the NEms was measured using a Zetasizer Ver. 7.13 (Malvern Instruments Pvt Ltd, UK). Emulsion stability was monitored over two weeks. For stability evaluation, samples were centrifuged at 2000 × g for 30 min every other day at 4 °C and 45 °C. After centrifugation, the samples were stored at room temperature (23–24 °C). At the end of two weeks, no phase separation or visible oil layer was observed (Özoğul et al. 2017).

### Apiary and bees

This study was conducted in 2024 at the apiary of the corresponding author located in Suvaran village, Muş Province, Türkiye (38.7714° N, 41.4306° E). The apiary consisted of 55 Langstroth-type hives, each equipped with plastic bottoms and pollen traps, and containing a single brood chamber with nine frames. Colonies selected for the experiment were headed by naturally mated Caucasian F1 hybrid queens introduced in June 2024 (Güneşdoğdu et al. 2022) and had sufficient sealed brood, typically occupying two frames per hive. These hives were carefully managed to ensure uniformity in colony strength, population size, brood quantity, and nutritional resources, minimizing variability among experimental units (Bava et al. 2025). No miticide treatments were applied for a five-month period prior to the experiment (from April to September) to ensure measurable baseline *Varroa* infestation levels. This allowed for a clear assessment of treatment-related reductions in mite load. High baseline infestation levels were intentionally allowed in order to evaluate treatment efficacy under severe *Varroa* pressure conditions. In September, the average temperature was 20.1 °C (above 0 °C and within the recommended 10–25 °C range for essential oil *Varroa* treatments; Akyol & Özkök, 2005) and the relative humidity was ~ 50% (below the < 60% level influencing the survival of *V. destructor*-infested bees; Annoscia et al. 2012) (Table S1; Turkish State Meteorological Service, 2024).

### Experimental design

In this experiment, essential oil (EO) concentrations were selected with minor modifications based on the values reported by Gamal Eldin et al. (2024). Three essential oils (thyme, peppermint, and eucalyptus) were evaluated across five treatment groups (50, 100, and 200 ppm, positive control, and negative control) using a factorial block design. Each dose was administered to the colonies by spraying.

Applications were performed using a hand-held sprayer at a rate of 5 mL per bee-covered frame, following the protocol described by Damiani et al. (2011) with slight modifications. Each colony consisted of nine bee-covered frames. Since 5 mL of formulation was applied per frame, the total spray volume per colony per treatment was approximately 45 mL. Accordingly, the total amount of essential oil delivered per colony per application was approximately 2.25 mg, 4.5 mg, and 9 mg for the 50, 100, and 200 ppm treatments, respectively. The experiment involved 55 honeybee colonies naturally infested with *Varroa destructor*. No *Varroa* control measures had been applied to the colonies for approximately five months prior to the start of the experiment.

The experimental colonies included three essential oils evaluated at three concentration levels (50, 100, and 200 ppm), with five colonies assigned to each oil–concentration combination (total: 45 colonies). In addition, one positive control group treated with amitraz (5 colonies) and one negative control group (5 colonies) were included. Amitraz (N-methyl-bis(2,4-xylyliminomethyl) amine) is a legally approved active substance in Türkiye to control *Varroa* mites. Colonies in the positive control group received strips containing 20 mg amitraz (Vamitrat-VA^®^), applied according to the method described by Özüiçli and Baykalır (2024).

All treatments were applied five times at 7-day intervals on days 0, 7, 14, 21, and 28. Untreated control groups were included to allow differentiation between treatment-induced mite mortality and natural mite declines resulting from colony defense mechanisms such as grooming and hygienic behavior. Throughout the trial, naturally falling mite counts were recorded weekly in control colonies. At the end of the study, the same final treatment protocol was applied to all colonies to ensure uniform colony management (Bava et al. 2025).

The *Varroa destructor* infestation level in adult worker bees was determined as the number of mites per 10 g of bees from samples containing approximately 100 worker bees (Karapetkovska-Hristova et al. 2024). Infestation levels were assessed using the sugar roll method described by Seven-Çakmak et al. (2017). Briefly, approximately 10 g of adult worker bees were placed in a glass jar with a fine-mesh lid, 20 g of powdered sugar was added, and the jar was gently shaken for two minutes to dislodge mites. The jar was then inverted onto a white sheet of paper, and dislodged mites were counted. After counting, bees were returned to their original colonies.

*Varroa* infestation levels were measured immediately before each treatment application and on days 0, 7, 14, 21, and 28. Infestation level was expressed as mites per 10 g of bees and calculated according to Güneşdoğdu and Abacı (2025) as follows:

1 $$\:Infestation\:Level\:\left(varroa\:per\:10\:g\:of\:bees\right)=\frac{Total\:EquationNumber\:of\:dislodged\:Varroa\times\:10}{Net\:weight\:of\:bees}$$

Treatment efficacy at each assessment day (7, 14, 21, and 28) was calculated according to Güneşdoğdu and Abacı (2025) as:

2 $$\:{\mathrm{\%}Efficacy}_{t}=\frac{{I}_{0}-{I}_{t}}{{I}_{0}}\times\:100$$

Where; $\:{I}_{0}$represents the infestation level prior to the first treatment application and $\:{I}_{t}$represents the infestation level measured at day $\:t$.

Fallen *Varroa* mites were additionally monitored using Vaseline-coated white paper placed in pollen drawers, a method widely employed for effective mite monitoring (Calderone and Lin 2003). Fallen mite counts were recorded on days 1, 3, and 5 following each treatment application, and these data are presented in Fig. 5. These measurements were independent of infestation assessments conducted on days 0, 7, 14, 21, and 28 (Fig. 4).

### Statistical Analyses

All statistical analyses were conducted for the sugar shake, mite drop, and *Varroa* infestation datasets. Before inferential analyses, the normality of the data was assessed using the Shapiro–Wilk test. As the obtained p-values were greater than 0.05, the assumption of normal distribution was considered satisfied. Temporal changes in the measured parameters were analyzed using one-way repeated measures analysis of variance (repeated measures ANOVA). The assumption of sphericity was evaluated using Mauchly’s test of sphericity. When the assumption was met (*p* > 0.05), standard results were interpreted. When the assumption was violated (*p* < 0.05), corrections were applied based on the epsilon (Ɛ) value: the Greenhouse–Geisser correction was used when Ɛ < 0.75, and the Huynh–Feldt correction was applied when Ɛ ≥ 0.75. When statistically significant differences were detected (*p* < 0.05), pairwise comparisons were performed using the Bonferroni adjustment to control for Type I error. Descriptive statistics are presented as mean ± standard error (SEM). All statistical analyses were performed using SPSS (SPSS Inc., Chicago, IL, USA). Graphical representations were generated using the SRplot online platform (Tang et al. 2023).

## Results

### Primary GC-MS component of essential oils

GC-MS analyses showed that thyme, eucalyptus, and peppermint essential oils (EOs) exhibited distinct profiles of major monoterpene compounds. Carvacrol was the predominant component in thyme oil (33.94%), followed by α-pinene, linalool, d-limonene, and p-cymene. Eucalyptus oil is mainly composed of eucalyptol (69.33%), with smaller amounts of d-limonene, α-pinene, p-cymene, and other minor monoterpenes. In peppermint oil, menthol was the primary constituent (39.70%), along with menthone isomers, neo-menthol, menthyl acetate, and other monoterpene derivatives (Table 2). Table 2 includes only the major compounds identified in the essential oils, whereas a more comprehensive chemical profile is provided in Table S2.

**Table 2:** Main chemical components and their quantity (%) of essential oils by GC-MS

No	Thyme Oil		Eucalyptus Oil		Peppermint Oil	
	Components	%	Components	%	Components	%
**1**	Carvacrol	33.94	Eucalyptol	69.33	Menthol, (±)-	39.70
**2**	1R-α-Pinene	21.36	D-Limonene	11.76	Isomenthone	20.90
**3**	Linalool	10.35	1R-α-Pinene	7.61	l-Menthone	8.20
**4**	D-Limonene	10.12	p-Cymene	7.13	Neo-Menthol	6.19
**5**	p-Cymene	5.87	.alpha.-Phellandrene	1.77	Menthol, acetate	6.12
**6**	.gamma.-Terpinene	2.56	.gamma.-Terpinene	0.84	Isopulegol	2.54
**7**	.beta.-Bisabolene	2.52	.beta.-Pinene	0.60	(-)-Carvone	2.20
**8**	Terpinen-4-ol	1.81	.beta.-Myrcene	0.55	.(±)-Pulegone	1.67
**9**	endo-Borneol	1.74	α-Terpinene	0.41	.alpha.-Terpineol	1.07
**10**	Thymol	1.06			Isopulegon	1.06

### Characterization of essential oil NEms

The physical properties of the prepared nanoemulsions were evaluated after formulation, revealing significant differences in droplet size among the three essential oil-based formulations (Table 3). The Thyme nanoemulsion (T-NEms) exhibited larger droplets, with an average diameter of approximately 123 nm. In contrast, the Peppermint nanoemulsion (P-NEms; 57.7 nm) and Eucalyptus nanoemulsion (E-Nems; 48.0 nm) produced significantly smaller droplets. Polydispersity index (PDI) values, which reflect the homogeneity of particle size distribution, support these findings. The PDI value of P-NEms was 0.142, indicating a narrow and uniform distribution, whereas E-NEms and T-NEms showed higher PDI values of 0.359 and 0.544, respectively, suggesting a broader particle size distribution, particularly for T-NEms. Viscosity values were similar for all NEms (0.8872 cP), indicating no notable differences in flow behavior among the nanoemulsions. Zeta potential analysis further supported the observed physical characteristics of the formulations.

**Table 3:** Physicochemical properties (Z-average, PDI, viscosity) and DLS-based particle size distribution of essential oil nanoemulsions

Parameters	Essential Oils		
	T-NEms	*P*-NEms	E-NEms
Z-avarege (d.nm)	123.1	57.73	47.99
PDI	0.544	0.142	0.359
Viscosity (cP)	0.8872	0.8872	0.8872
Zetasizer Analysis Images			
Zetasizer Analysis Images			
Zetasizer Analysis Images			

*PDI: polydispersity index, DLS measurements were performed using a Zetasizer instrument and size distribution is presented as intensity percentage.

### Nanoemulsion efficacy and mite dynamics

**Fig. 1:**
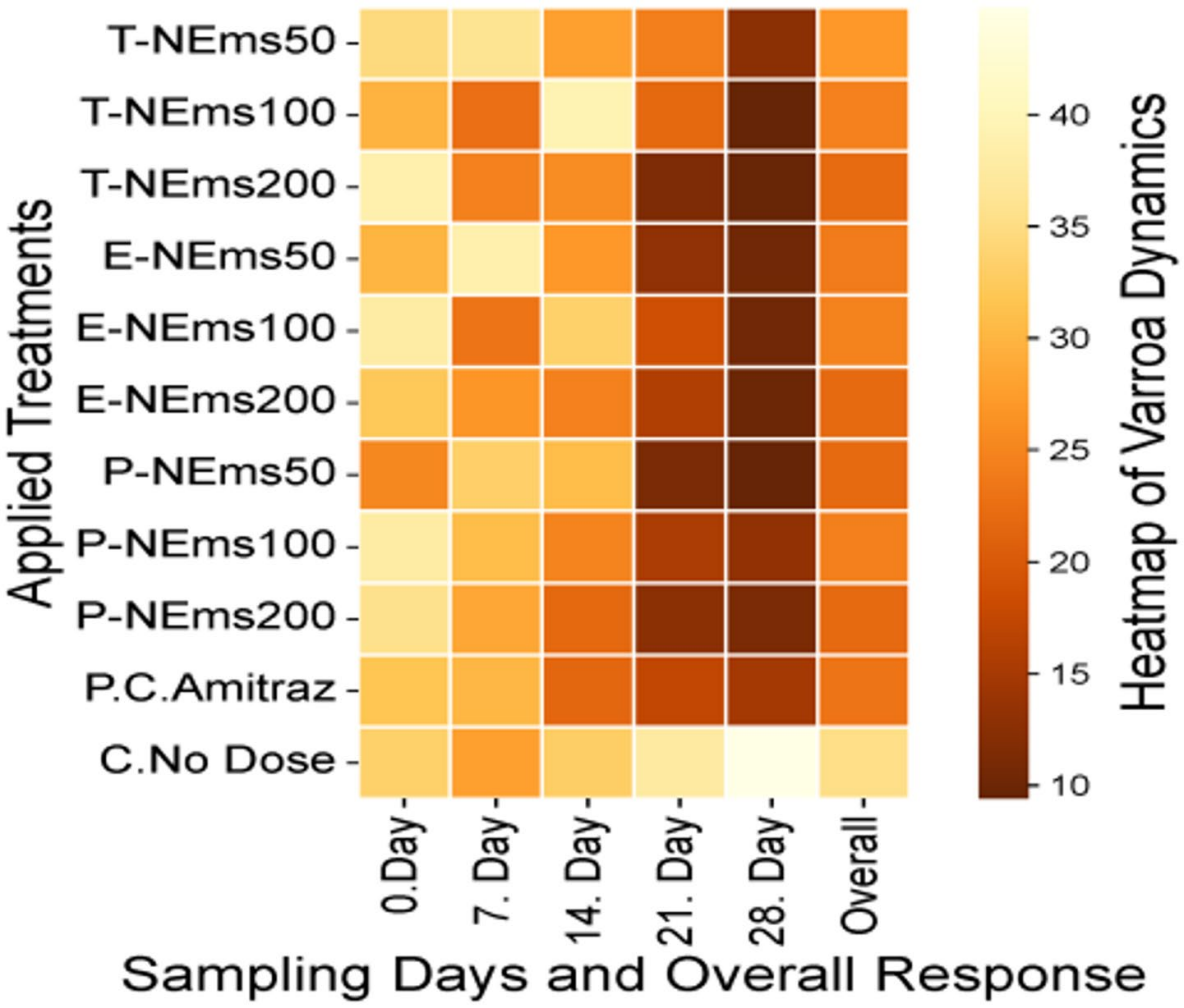
Mean ± SEM and statistical comparisons of Varroa infestation levels (mites per 10 g of bees) across treatments and sampling days (T-NEms: Thyme nanoemulsion; E-NEms: Eucalyptus nanoemulsion; P-NEms: Peppermint nanoemulsion; 50, 100, 200: Applied doses (ppm); PC-Amitraz: Positive control (amitraz); C-No Dose: Untreated control)

Varroa infestation levels, expressed as the number of mites per 10 g of bees and determined by the sugar shake method, are summarized in Table S3 (mean ± SEM, *n* = 5) and illustrated in Fig. 1. Baseline infestation levels (Day 0) ranged from 29.8 ± 6.37 to 38.8 ± 2.48 mites per 10 g of bees among treated groups, with no significant differences between treatments at the start of the experiment (*p* > 0.05). A repeated measures ANOVA revealed significant effects of time, treatment, and their interaction (*p* < 0.05). No significant differences among treatment groups were detected on Days 0, 7, or 14 (*p* > 0.05). However, significant treatment effects emerged on Days 21 and 28 (*p* < 0.05). Within-treatment comparisons demonstrated that most nanoemulsion formulations significantly reduced infestation levels by Day 28 compared to baseline values (*p* < 0.05). The greatest reduction was observed in the T-NEms200 group, where infestation decreased from 38.8 ± 2.48 to 9.6 ± 1.21 mites per 10 g of bees by Day 28 (75.42% mean reduction). Similar reductions were observed in E-NEms100 (37.8 ± 3.15 to 10.4 ± 1.08) and P-NEms200 (35.6 ± 3.17 to 11.2 ± 0.86). In contrast, infestation levels in the untreated control group increased significantly over time, rising from 33.4 ± 3.59 to 44.8 ± 2.50 mites per 10 g of bees by Day 28 (*p* < 0.05). The positive control (amitraz) reduced infestation from 31.8 ± 1.62 to 14.8 ± 1.28 by Day 28, corresponding to a 53.12% mean reduction. Pairwise comparisons indicated that by Day 28, all nanoemulsion treatments achieved significantly lower infestation levels compared with the untreated control group (*p* < 0.05), while several nanoemulsion formulations demonstrated comparable or greater reductions than the amitraz treatment.

**Fig. 2:**
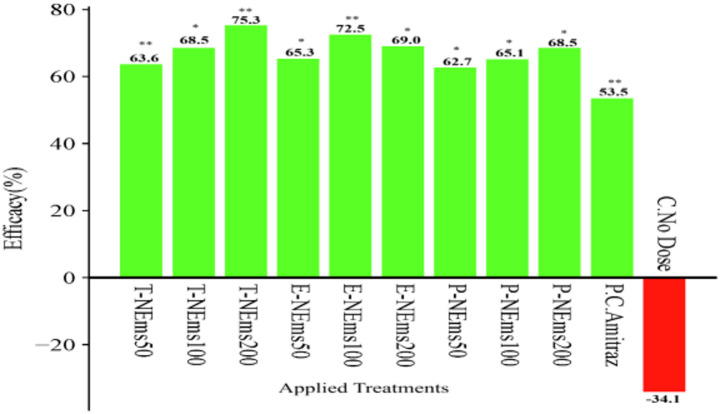
Treatment efficacy (%), calculated as the percentage reduction in mean Varroa infestation levels (mites per 10 g of bees) from Day 0 to Day 28. Asterisks indicate significant differences from baseline infestation levels (paired t-test; *p < 0.05, **p < 0.001). Abbreviations: T-NEms, thyme nanoemulsion; E-NEms, eucalyptus nanoemulsion; P-NEms, peppermint nanoemulsion; PC-Amitraz, positive control; C-No Dose, untreated control. Efficacy values are presented for descriptive purposes only and were calculated from infestation data; statistical analyses were performed on infestation levels rather than on efficacy percentages

Nanoemulsion treatments showed higher efficacy (%), calculated as the percentage reduction in Varroa infestation levels (mites per 10 g of bees) from Day 0 to Day 28, compared to the positive control amitraz (53.5%). Efficacy values ranged from 62.7% to 75.3% across all nanoemulsion doses. The highest efficacy was observed in the T-NEms200 group (75.3%). In contrast, Varroa infestation levels in untreated control colonies increased by 34.1% over the same period, indicating the absence of mite suppression in the control group (Fig. 2).

**Fig. 3:**
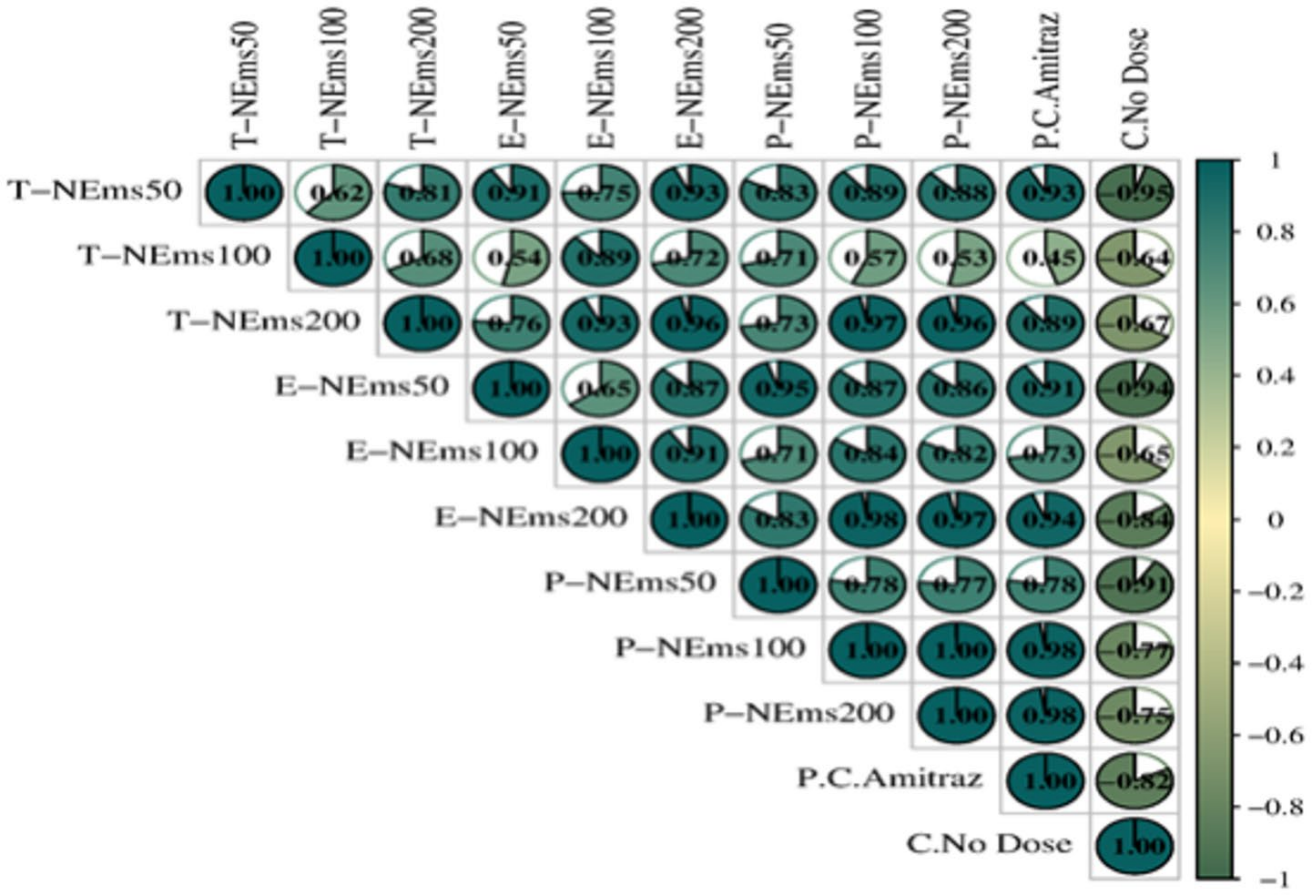
Correlation matrix showing relationships among *Varroa destructor* infestation levels across nanoemulsion treatments, the positive control (amitraz), and untreated colonies. Positive correlations indicate similar temporal trends in infestation levels, whereas negative correlations reflect the opposite trend observed in untreated colonies. Correlation analysis illustrates pattern similarity and does not imply treatment efficacy or dose-dependent effects. (**T-NEms: Thyme nanoemulsion; E-NEms: Eucalyptus nanoemulsion; P-NEms: Peppermint nanoemulsion; 50, 100, 200: Applied doses (ppm); PC-Amitraz: Positive control (amitraz); C-No Dose: Untreated control)

The correlation matrix (Fig. 3) was included to assess whether different essential oil nanoemulsion formulations produced comparable temporal suppression patterns of *Varroa* infestation. Strong positive correlations among nanoemulsion treatments (*r* = 0.71–1.00) indicate consistent infestation dynamics across essential oil types and doses, suggesting that the primary difference among treatments lies in magnitude of reduction rather than the overall pattern of decline. Negative correlations observed in the untreated control group confirm its divergent infestation trajectory. While efficacy and dose-response effects were statistically evaluated using repeated-measures ANOVA, the correlation analysis provides complementary insight into the consistency of temporal treatment dynamics.

### Fallen mite counts

**Fig. 4:**
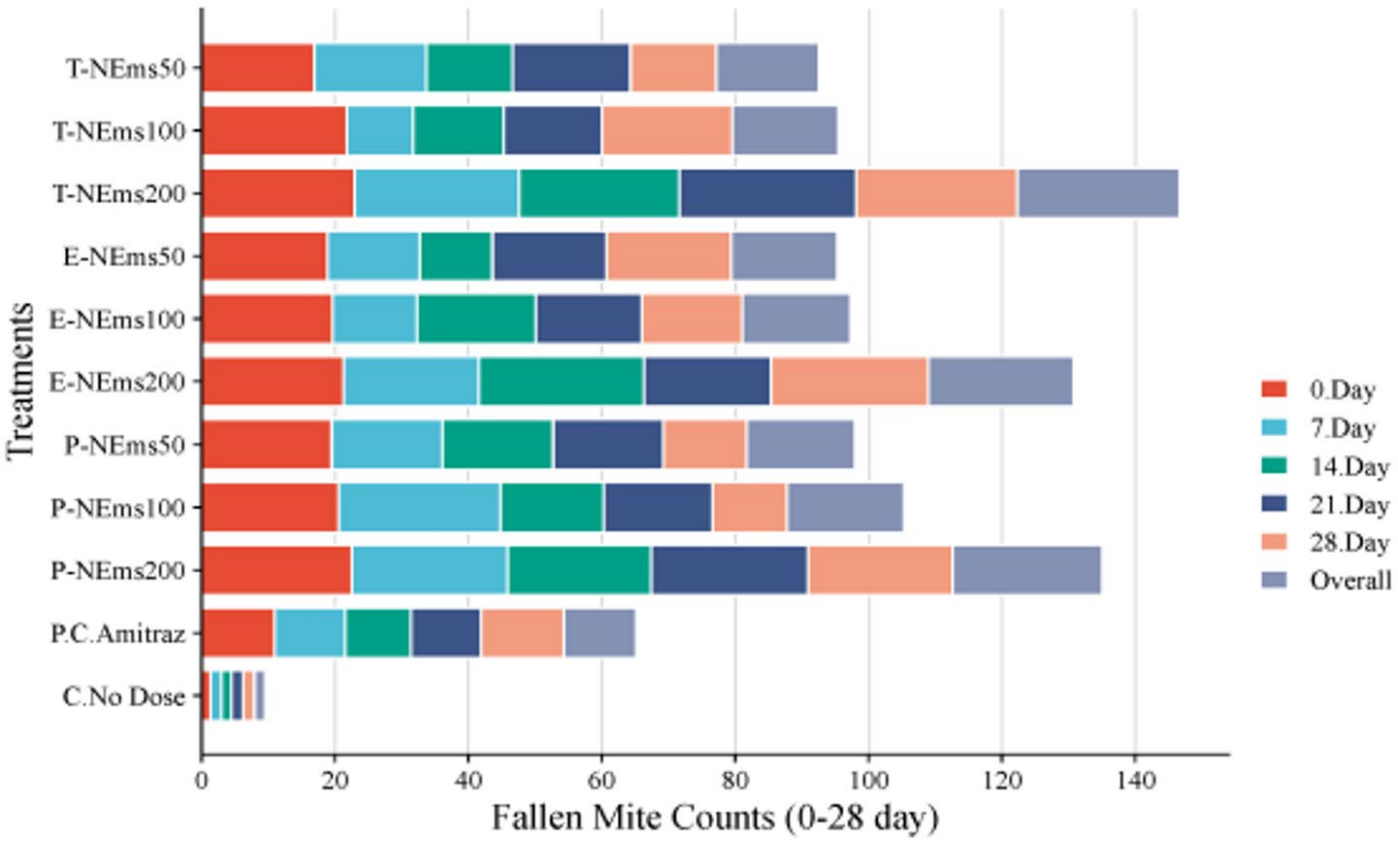
Daily fallen mite counts (Mean ± SEM) recorded at 0, 7, 14, 21, and 28 days after treatment across all experimental groups. (**T-NEms: Thyme nanoemulsion; E-NEms: Eucalyptus nanoemulsion; P-NEms: Peppermint nanoemulsion; 50, 100, 200: Applied doses (ppm); PC-Amitraz: Positive control (amitraz); C-No Dose: Untreated control)

Cumulative fallen mite counts recorded over the 28-day experimental period are presented in Fig. 4 and Table S4. Fallen mites were collected at 1, 3, and 5 days following each weekly treatment, and counts were summed to obtain weekly cumulative mite fall values for Days 0, 7, 14, 21, and 28. Repeated measures ANOVA revealed a highly significant effect of treatment (*P* < 0.001) and a significant Treatment × Day interaction (*P* = 0.007), indicating that mite fall dynamics differed among treatments over time. The overall effect of day was not statistically significant (*P* = 0.075). Across all sampling days, the 200 ppm nanoemulsion treatments (T-NEms200, E-NEms200, and P-NEms200) consistently produced significantly higher cumulative mite fall compared with lower doses and the positive control. Amitraz induced moderate mite fall but remained significantly lower than the highest nanoemulsion doses. In contrast, the untreated control group exhibited minimal and stable natural mite fall throughout the study period, confirming that the elevated mite drop observed in treated colonies was treatment-induced rather than spontaneous.

**Fig. 5:**
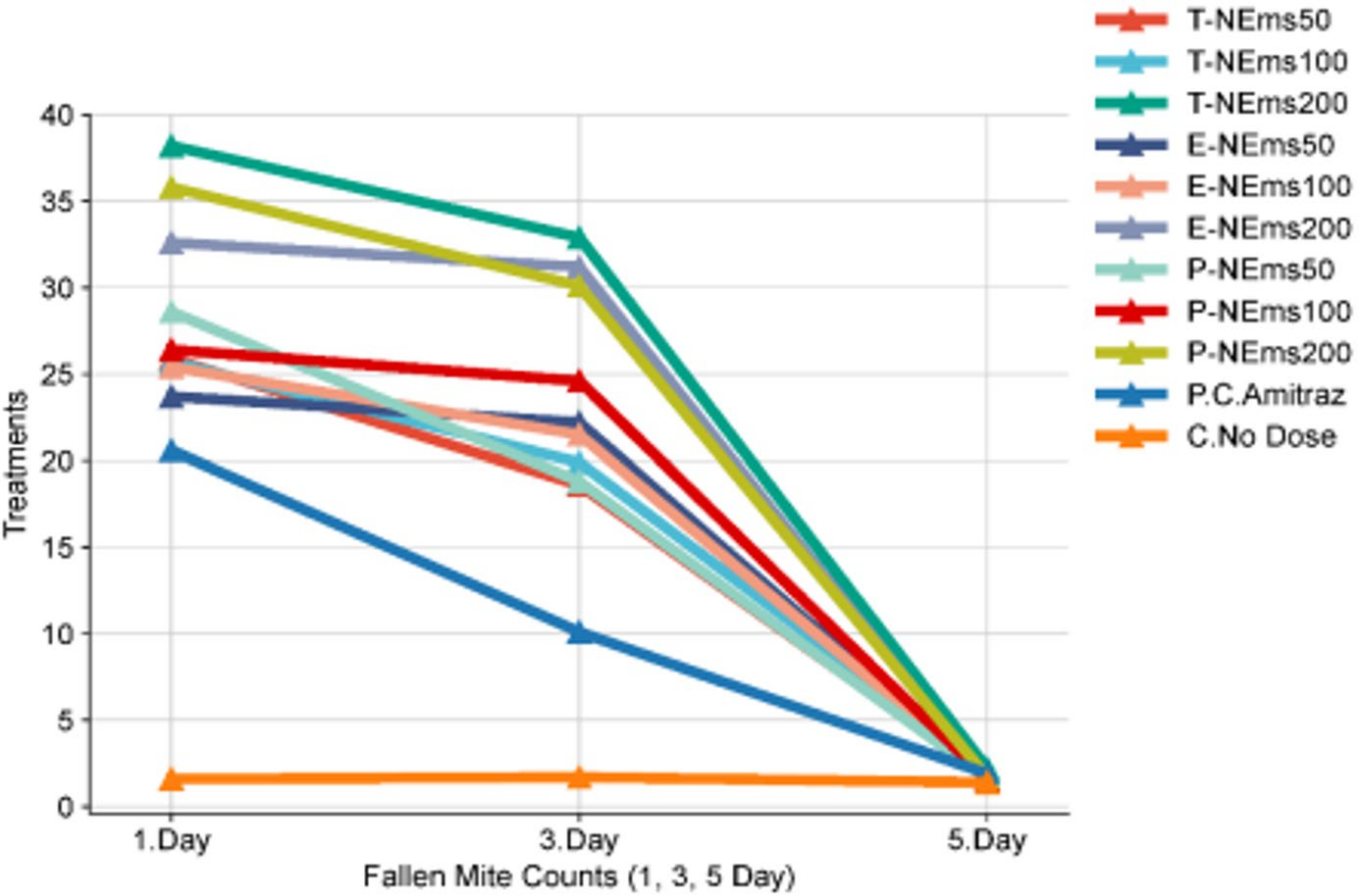
Mean numbers of fallen *Varroa destructor* mites recorded 1, 3, and 5 days after treatment, averaged across all sampling periods (0–28 days). Statistical comparisons among treatments and sampling days are presented in Table S5. (**T-NEms: Thyme nanoemulsion; E-NEms: Eucalyptus nanoemulsion; P-NEms: Peppermint nanoemulsion; 50, 100, 200: applied doses, ppm; PC-Amitraz: positive control; C-No Dose: untreated control)

Figure 5 illustrates the short-term changes in fallen *Varroa destructor* counts following treatment, commonly referred to as “acute mite fall.” As shown in the figure, the T-NEms200, E-NEms200, and P-NEms200 groups exhibited higher mite fall on days 1 and 3 compared to the lower-dose treatments, reflecting differences in early post-treatment mite dynamics. Across all treatments, mite fall values decreased markedly by day 5, reaching similarly low levels. This pattern suggests that the majority of treatment-related mite fall occurred within the first 72 h after application, while subsequent counts reflected reduced short-term activity rather than continued treatment effects. The amitraz positive control showed intermediate mite fall on days 1 and 3 compared with the nanoemulsion treatments. Statistical analysis based on fallen mite counts confirmed significant effects of treatment (*P* < 0.001), time (*P* < 0.001), and the treatment × time interaction (*P* < 0.001), as detailed in Table S5.

## Discussion

The *Varroa destructor* mite is a major problem for beekeeping worldwide, and extensive research has been conducted and continues to develop effective control strategies. Although creating genetically resistant bee lines (Guichard et al. 2020) appears to be an effective solution, beekeepers prefer immediate control methods because genetic selection is a long-term process. Chemical treatments, while fast-acting, pose risks to both bee and human health. In contrast, essential oils offer a more sustainable treatment method. Their complex structures make it difficult for mites to develop resistance (Bava et al. 2025).

Thyme oil is generally obtained from *Thymus vulgaris* L., but it can also be derived from other species of the *Thymus* genus (Radev 2022). Recent studies have demonstrated that the chemical composition of essential oils shows substantial variability influenced by the vegetative cycle, genetic background, chemotype, and environmental conditions, as well as differences among species, subspecies, and extraction-related factors (Benomari et al. 2023). Thyme essential oil is a complex mixture containing more than 70 components, with thymol generally reported as the dominant compound (56–60%), accompanied by p-cymene, γ-terpinene, α-pinene, caryophyllene, and other constituents whose relative proportions vary with plant origin and environmental conditions (Hossain et al. 2022). Variability in thyme oil composition has also been documented among species and geographic origins; for example, Bisrat et al. (2022) reported carvacrol, thymol, and p-cymene as the main constituents of *Thymus schimperi* oil and demonstrated acaricidal activity of thymol and carvacrol. Similarly, Hýbl et al. (2021) identified thymol and p-cymene as major components of thyme oil, while peppermint oil was characterized by limonene, menthol, and other monoterpenes. In the present study, GC–MS analysis (Table 2) showed that carvacrol, eucalyptol, and menthol were the predominant constituents of thyme, eucalyptus, and peppermint oils, respectively, which is consistent with the reported compositional variability of these oils. Previous studies have shown that nanoemulsion-based formulations may alter the relative proportions of essential oil constituents, often increasing the prevalence of major bioactive compounds such as menthol or carvacrol (Özoğul et al. 2017; Youssef and Abdelmegeed 2021). In this context, the biological effects observed in the present study are best interpreted in relation to the dominance of these bioactive components and their effective delivery through the nanoemulsion system.

The principal compound responsible for the acaricidal activity of eucalyptus oil against *Varroa* mites is eucalyptol (1,8-cineole) (Atmani-Merabet et al. 2025). Previous studies have reported droplet diameters of eucalyptus nanoemulsions in the nanoscale range, including approximately 70–120 nm (Sharma et al. 2021; Cai et al. 2023; Barros et al. 2025). Similarly, nanoemulsions prepared from *Thymus* species—rich in thymol, carvacrol, p-cymene, and linalool—have been reported to exhibit droplet sizes generally between 70 and 130 nm (Özoğul et al. 2017; Wan et al. 2019; Elshamy et al. 2020; Mansouri et al. 2021; Moazeni et al. 2021; Abdelhamed et al. 2022; Doghish et al. 2023; Nasra et al. 2024). For peppermint oil, whose main active constituents are menthol and carvone, reported droplet diameters range from approximately 50 to 200 nm depending on species and formulation (Zamaniahari et al. 2022; El-Naggar et al. 2023).

Collectively, these studies indicate that essential oil nanoemulsions are commonly formulated within the 50–200 nm range, which is considered optimal for stability and biological applications (Tadros et al. 2004; Solans and Solé 2012; Jaiswal et al. 2015; Liang et al. 2022). Smaller droplet diameters increase surface area and may facilitate more efficient contact between active compounds and the target organism. However, droplet size alone does not determine stability or biological efficacy; it is one of several contributing factors (Liang et al. 2022).

In the present study, the droplet diameters obtained for all formulations fell within the recommended nanoscale range. This size distribution likely contributed to the kinetic stability of the nanoemulsions and may have enhanced the interaction of bioactive compounds particularly phenolic constituents such as thymol and carvacrol with *Varroa* mites. Nevertheless, based on our data, the observed biological activity cannot be attributed solely to phenolic content, but rather to the combined effects of chemical composition and physicochemical properties of the nanoemulsions.

The effectiveness of *M. piperita* and *T. vulgaris* oils observed in this study is consistent with the known chemical profile of Lamiaceae species, which are rich in monoterpene-containing essential oils with documented acaricidal activity against *V. destructor* (Ramzi et al. 2017). Although *Eucalyptus globulus* belongs to the Myrtaceae family, its essential oil also demonstrates significant potential for *Varroa* control due to comparable bioactive constituents (Kouache et al. 2017; Aglagane et al. 2021; Bisrat et al. 2022). Laboratory and field studies similarly report strong mite mortality following peppermint and thyme applications, particularly in nano-formulations (Hýbl et al. 2021; El Roby et al. 2023; Gamal Eldin et al. 2024).

In agreement with reports highlighting the efficacy of carvacrol-rich oils (Ramzi et al. 2017; Khajehali et al. 2023), our results show that T-NEms200 exhibited the highest acaricidal activity among the tested formulations. This higher efficacy may be attributed to the combined effect of its carvacrol-rich composition and the nanoemulsion formulation, which likely enhanced dispersion and contact with mites. Smaller droplet sizes increase surface area and facilitate more efficient interaction between active compounds and target organisms, thereby improving biological activity (Leong et al. 2009). The moderate efficacy observed at other doses is also consistent with literature indicating variable commercial performance of essential-based products (Bava et al. 2024; Reyna-Fuentes et al. 2025). While increasing doses have been associated with greater mite fall in several studies (Conti et al. 2020), differences in application timing and formulation may explain variations in efficacy patterns across experiments (Bakar et al. 2017 tükoğlu et al., 2012).

Several studies have reported that mite populations increase substantially in untreated control groups (Narciso et al. 2024; Raza et al. 2024), a trend also observed in earlier investigations (Akyol and Yeninar 2008). This supports the biological relevance of the reductions recorded in our treated groups. Moreover, consistent with previous findings that the most pronounced mite decline occurs shortly after essential oil application (Ramzi et al. 2017; Güneşdoğdu and Abacı 2025), the highest reduction in our study was detected on the first day following treatment. The highest mite reduction observed on the first day after application, followed by lower mite counts on days 3 and 5, indicates that the treatments primarily exert a rapid knockdown effect, with limited persistence over time. Reports of variable amitraz efficacy, possibly linked to resistance development (Aydın and Girişgin 2010; Adjlane and Haddad 2017; Koç et al. 2021; Hernández-Rodríguez et al. 2021), further contextualize the 53.5% efficacy observed for the positive control in our study.

Although essential oils are increasingly evaluated in terms of both potential risks (Da Silva et al. 2020) and benefits for honey bees (Conti et al. 2020), and some studies report no adverse effects on colony health (Narciso et al. 2024), safety considerations should extend beyond the active ingredients to include formulation components. Certain surfactants have been reported to exhibit toxicity to honey bees and may influence colony health depending on concentration and exposure route (Shannon et al. 2023). Therefore, careful selection of formulation excipients is essential when developing nanoemulsion-based miticides. Further targeted research is needed to clarify the long-term biopesticide performance and safety profile of both active compounds and carrier systems (Gostin and Popescu 2023). Overall, our findings align with the growing body of evidence supporting plant-derived compounds as promising alternatives in *Varroa* management (Ruffinengo et al. 2014). From a practical perspective, essential oil nanoemulsions offer several advantages, including relatively low production costs and the potential for valorization of agro-industrial by-products. For example, nanoemulsion formulations derived from plant materials such as grapefruit peel have been reported as cost-effective and sustainable alternatives with potential for large-scale production (Çiçek et al. 2024). However, certain limitations should be considered. The application of such formulations may be time-consuming, particularly in strong colonies where achieving uniform distribution can be challenging. In addition, although essential oils are generally regarded as environmentally friendly, repeated applications may be required due to their short-term knockdown effect, which could raise concerns regarding potential residue accumulation and labor costs. Therefore, further studies are needed to optimize application strategies and evaluate long-term feasibility under practical beekeeping conditions.

Field efficacy of miticidal treatments is strongly influenced by seasonal brood dynamics and climatic conditions. *Varroa destructor* reproduction depends on capped brood availability, and seasonal variation in brood abundance has been shown to significantly shape autumn infestation levels (Smoliński et al. 2021; Nürnberger et al. 2019). Raised spring and autumn temperatures may reinforce mite population growth through effects on bee abundance and brood production (Smoliński et al. 2021), while broader seasonal population patterns further highlight the ecological sensitivity of *Varroa* dynamics (Jack et al. 2023). In addition, drifting and robbing behavior among neighboring colonies can facilitate horizontal mite transmission and reinfestation, particularly in dense apiaries (Peck and Seeley 2019). Because the present study was conducted within a single seasonal window and a single apiary location, the reported efficacy values should be interpreted within this ecological and spatial context. In addition, inter-colony transmission of mites may have influenced the observed mite reduction levels. Drifting and robbing behaviors are well-documented mechanisms facilitating mite movement between colonies, particularly in apiary settings, and may contribute to reinfestation or fluctuations in mite populations during field experiments (Peck and Seeley 2019; Goodwin et al. 2006). Notably, rapid increases in mite levels in neighboring colonies have been associated with robbing of heavily infested or collapsing colonies, which can override spatial separation effects and complicate interpretation of treatment efficacy. Furthermore, mite quantification based on sugar shake methods may underestimate actual infestation levels due to incomplete mite recovery, as this method has been reported to detect fewer mites compared to alcohol wash under certain conditions (Owen et al., 2022). Therefore, the efficacy values reported in the present study should be interpreted with caution, as both biological factors (mite drift and reinfestation) and methodological constraints may have influenced the measured mite reduction.

A limitation of this study is the absence of a carrier-only control group (Tween 80, ethanol, and water). Although these components are generally considered inert or of low toxicity at the concentrations commonly used in nano-formulations (Islam et al. 2020), their potential physical contribution to mite mortality cannot be completely excluded. Therefore, the observed efficacy should be interpreted as the overall performance of the essential oil-based nanoemulsion rather than the isolated biochemical effect of the essential oils. Future studies should include a carrier control to more clearly differentiate formulation effects.

A further limitation of the present study is that potential acute or chronic effects of the nanoemulsion treatments on adult bees, brood development, queen performance, or residue levels in hive products were not evaluated. Therefore, the findings should be interpreted strictly in terms of acaricidal efficacy. Comprehensive safety assessments represent a critical next step to validate the practical applicability of essential oil-based nanoemulsions under field conditions.

## Conclusion

This study demonstrates that nanoemulsions formulated with thyme, peppermint, and eucalyptus essential oils exhibit acaricidal activity against *Varroa destructor* under the conditions tested. The nanoscale droplet size of the formulations likely contributed to their physicochemical stability and may have enhanced the interaction of active compounds with mites. Among the tested formulations, differences in efficacy were observed depending on oil type and concentration. While these findings indicate that essential oil-based nanoemulsions have potential as alternative tools in *Varroa* management, the results are limited to the experimental conditions of the present study. Further large-scale and long-term field trials are necessary to evaluate their safety for brood stages, possible residue formation in bee products, and overall colony-level effects before practical application can be recommended.

## Supplementary Information

Below is the link to the electronic supplementary material.

Supplementary material 1 (DOCX 34.9 kb)

Supplementary material 2 (DOCX 33.3 kb)

Supplementary material 3 (PDF 149.7 kb)

Supplementary material 4 (CSV 5.1 kb)

## Data Availability

Data will be made available on request.
