# Supplementary material for: Colony-Level Efficacy of Mentha piperita, Thymus vulgaris and Eucalyptus globulus Essential Oil Nanoemulsions Against Varroa destructor
Source: Exp Appl Acarol. 2026 May 5;96(4):50. doi: 10.1007/s10493-026-01141-y (PMC13144229; doi:10.1007/s10493-026-01141-y)
Supplement: Supplementary file 1 — Supplementary material 1 (DOCX 34.9 kb) [file 10493_2026_1141_MOESM1_ESM.docx]

**Table S1.** Climatic data of the trial location

| **Climatic Characteristics** | **September** | **October** | **November** | **December** |
| --- | --- | --- | --- | --- |
| **Monthly Average Temperature (°C)** | 20.9 | 13.0 | 5.3 | -0.9 |
| **MonthlyAverage Min. Temperature (°C)** | 14.7 | 7.2 | 1.5 | -3.0 |
| **MonthlyAverage Max. Temperature (°C)** | 28.0 | 20.4 | 9.9 | 1.8 |
| **Average Relative Humidity (%)** | 46.6 | 49.6 | 75.7 | 83.8 |
| **Avg. Precipitation (mm=kg/m²)** | 40.2 | 43.8 | 61.4 | 2.4 |
| **Monthly Average Pressure (hPa)** | 867.6 | 870.9 | 872.2 | 874.7 |

*hPa: Hectopascal, °C: centigrade degree, mm=kg/m²: millimeter=kilogram/square meter, %: percentage

**Table S2.** Main Chemical Compound and and Their Quantity (%) of EOs by GC-MS.

|  |  | **Thyme Oil** | | **Eucalyptus Oil** | | **Peppermint Oil** | |
| --- | --- | --- | --- | --- | --- | --- | --- |
| **No** |  | **Components** | **%** | **Components** | **%** | **Components** | **%** |
| **1** |  | Carvacrol | 33.94 | Eucalyptol | 69.33 | Menthol, (±)- | 39.70 |
| **2** |  | 1R-α-Pinene | 21.36 | D-Limonene | 11.76 | Isomenthone | 20.90 |
| **3** |  | Linalool | 10.35 | 1R-α-Pinene | 7.61 | l-Menthone | 8.20 |
| **4** |  | D-Limonene | 10.12 | p-Cymene | 7.13 | Neo-Menthol | 6.19 |
| **5** |  | p-Cymene | 5.87 | .alpha.-Phellandrene | 1.77 | Menthol, acetate | 6.12 |
| **6** |  | .gamma.-Terpinene | 2.56 | .gamma.-Terpinene | 0.84 | Isopulegol | 2.54 |
| **7** |  | .beta.-Bisabolene | 2.52 | .beta.-Pinene | 0.60 | (-)-Carvone | 2.20 |
| **8** |  | Terpinen-4-ol | 1.81 | .beta.-Myrcene | 0.55 | .(±)-Pulegone | 1.67 |
| **9** |  | endo-Borneol | 1.74 | α-Terpinene | 0.41 | .alpha.-Terpineol | 1.07 |
| **10** |  | Thymol | 1.06 |  |  | Isopulegon | 1.06 |
| **11** |  | cis-Sabinene hydrate | 0.36 |  |  | D-Limonene | 0.68 |
| **12** |  | Camphene | 0.84 |  |  | 3-Octanol, 3,7-dimethyl- | 0.25 |
| **13** |  | Naphthalene, 1,2,3,4,4a,5,6,7-octahydro-4a-methyl- | 0.15 |  |  | .p-Menthan-4-ol | 0.29 |
| **14** |  | Terpinolene | 0.22 |  |  | (-)-.beta.-Bourbonene | 0.50 |
| **15** |  | 1-Octen-3-ol | 0.23 |  |  | p-Menthan-1-ol, trans- | 1.02 |
| **16** |  | Sabinene hydrate | 0.21 |  |  | 5-Tridecene, (Z)- | 0.31 |
| **17** |  | Camphor | 0.11 |  |  | 7-Octen-2-ol, 2,6-dimethyl- | 0.40 |
| **18** |  | .beta.-Pinene | 0.42 |  |  | Isopregol | 0.92 |
| **19** |  | trans-Sabinene hydrate | 0.15 |  |  | (-)-trans-Pinane | 0.49 |
| **20** |  | Linalyl acetate | 0.21 |  |  | Caryophyllene | 0.59 |
| **21** |  | .tau.-Cadinol | 0.18 |  |  | Levomenthol | 0.83 |
| **22** |  | Caryophyllene | 1.28 |  |  | p-Menthan-1-ol | 0.76 |
| **23** |  | β-Terpineol | 0.15 |  |  | .(±)-Lavandulol | 0.94 |
| **24** |  | .alpha.-Terpineol | 0.83 |  |  | Piperitone | 0.93 |
| **25** |  | .beta.-Myrcene | 1.43 |  |  | 1-Decanol | 0.59 |
| **26** |  | Aromandendrene | 0.12 |  |  |  |  |
| **27** |  | (-)-Carvone | 0.16 |  |  |  |  |
| **28** |  | α-Terpinene | 0.94 |  |  |  |  |
| **29** |  | .(-)-β-Cadinene | 0.13 |  |  |  |  |
| **30** |  | .gamma.-Muurolene | 0.11 |  |  |  |  |
| **31** |  | Carvacryl acetate | 0.15 |  |  |  |  |
| **32** |  | Caryophyllene oxide | 0.17 |  |  |  |  |
| **33** |  | 2-Methyl-5-(propan-2-ylidene)cyclohexane-1,4-diol | 0.11 |  |  |  |  |
| **Total** |  | 100 | | | | | |

**Table S3**. Temporal Changes in Varroa Infestation Rates in Honeybee Colonies Treated with Thyme, Peppermint, and Eucalyptus Oils (Mean±SEM)

| **n** | **Treatment** | **0 Day** | **7 Day** | **14 Day** | **21 Day** | **28 Day** | **Overall** | P < 0.05 |
| --- | --- | --- | --- | --- | --- | --- | --- | --- |
| **5** | **T-NEms50** | 34.6 ± 3.87 | 36.2 ± 3.55 | 27.8 ± 4.21 | 24.2 ± 7.46 | 12.6 ± 2.42 | 27.08±2.56 |  |
| **5** | **T-NEms100** | 29.8 ± 6.37 | 22.6 ± 3.61 | 39.4 ± 5.20 | 21.8 ± 4.05 | 9.4 ± 1.03 | 24.60±2.71 |  |
| **5** | **T-NEms200** | 38.8 ± 2.48 | 24.6 ± 4.20 | 25.8 ± 2.61 | 11.6 ± 1.94 | 9.6 ± 1.20 | 22.08±2.43 |  |
| **5** | **E-NEms50** | 30.0 ± 3.47 | 38.8 ± 3.65 | 27.0 ± 4.74 | 13.0 ± 2.70 | 10.4 ± 1.07 | 23.84±2.57 |  |
| **5** | **E-NEms100** | 37.8 ± 3.15 | 23.2 ± 3.40 | 33.4 ± 5.70 | 18.6 ± 2.77 | 10.4 ± 1.07 | 24.68±2.49 |  |
| **5** | **E-NEms200** | 32.2 ± 6.16 | 26.8 ± 6.63 | 24.6 ± 6.09 | 15.8 ± 1.91 | 10.0 ± 2.53 | 21.88±2.63 |  |
| **5** | **P-NEms50** | 25.2 ± 4.49 | 33.2 ± 4.40 | 30.8 ± 4.91 | 11.2 ± 2.06 | 9.4 ± 0.87 | 21.96±2.52 |  |
| **5** | **P-NEms100** | 37.8 ± 3.26 | 30.8 ± 4.78 | 24.8 ± 2.85 | 15.6 ± 1.50 | 13.2 ± 1.39 | 24.44±2.25 |  |
| **5** | **P-NEms200** | 35.6 ± 3.17 | 28.6 ± 5.21 | 21.8 ± 4.05 | 12.6 ± 1.87 | 11.2 ± 0.86 | 21.96±2.35 |  |
| **5** | **P.C.Amitraz** | 31.8 ± 1.62 | 30.2 ± 5.01 | 21.6 ± 3.98 | 17.4 ± 1.17 | 14.8 ± 1.28 | 23.16±1.86 |  |
| **5** | **C.No Dose** | 33.4 ± 3.59 | 27.8 ± 5.09 | 33.0 ± 6.26 | 37.6 ± 2.38 | 44.8 ± 2.50 | 35.32±2.08 |  |
| **P Values** | | T | P > 0.05; 0.796 | | | | | |
|  |  | Ds | P > 0.05; 0.487 | | | | | |
|  |  | T x Ds | P > 0.05; 0.553 | | | | | |

*T: treatment, Ds: Dose (50, 100, and 200 ppm), x: interaction; T-NEms: Thyme nanoemulsion; E-NEms: Eucalyptus nanoemulsion; P-NEms: Peppermint nanoemulsion; 50, 100, 200: Applied doses (ppm); PC-Amitraz: Positive control (amitraz); C-No Dose: Untreated control.

**Table S4.** Daily fallen mite counts (Mean±SEM) recorded at 0, 7, 14, 21, and 28 days after treatment across all experimental groups.

| **n** | **Treatment** | **0 Day** | **7 Day** | **14 Day** | **21 Day** | **28 Day** | **Overall** | P < 0.05 |
| --- | --- | --- | --- | --- | --- | --- | --- | --- |
| **5** | **T-NEms50** | 16.87 ± 1.08 | 16.80 ± 1.64 | 12.93 ± 3.37 | 17.67 ± 3.31 | 12.87 ± 1.97 | 15.43±2.43b |  |
| **5** | **T-NEms100** | 21.80 ± 2.98 | 9.87 ± 2.83 | 13.60 ± 2.97 | 14.73 ± 3.00 | 19.60 ± 1.40 | 15.92±3.15 b |  |
| **5** | **T-NEms200** | 22.87 ± 2.06 | 24.67 ± 2.12 | 24.00 ± 1.69 | 26.47 ± 1.47 | 24.20 ± 1.71 | 24.44±1.75a |  |
| **5** | **E-NEms50** | 18.87 ± 2.04 | 13.80 ± 1.51 | 10.93 ± 3.90 | 17.07 ± 2.74 | 18.73 ± 1.93 | 15.88±2.73b |  |
| **5** | **E-NEms100** | 19.60 ± 1.88 | 12.67 ± 3.15 | 17.80 ± 2.63 | 15.87 ± 2.83 | 15.07 ± 3.26 | 16.20±2.77b |  |
| **5** | **E-NEms200** | 21.27 ± 1.59 | 20.27 ± 1.14 | 24.73 ± 1.02 | 19.07 ± 1.96 | 23.60 ± 2.12 | 21.79±1.77a |  |
| **5** | **P-NEms50** | 19.53 ± 2.21 | 16.60 ± 2.34 | 16.60 ± 1.28 | 16.47 ± 1.53 | 12.53 ± 1.42 | 16.35±1.94b |  |
| **5** | **P-NEms100** | 20.47 ± 2.41 | 24.27 ± 2.19 | 15.47 ± 2.90 | 16.27 ± 2.14 | 11.20 ± 2.26 | 17.53±2.99b |  |
| **5** | **P-NEms200** | 22.47 ± 0.44 | 23.40 ± 1.31 | 21.53 ± 2.53 | 23.47 ± 1.32 | 21.73 ± 1.34 | 22.52±1.45a |  |
| **5** | **P.C.Amitraz** | 10.93 ± 1.19 | 10.60 ± 0.95 | 9.93 ± 0.81 | 10.47 ± 1.16 | 12.40 ± 2.21 | 10.87 ± 1.30c |  |
| **5** | **C.No Dose** | 1.33 ± 0.18 | 1.67 ± 0.45 | 1.53 ± 0.36 | 1.73 ± 0.48 | 1.67 ± 0.18 | 1.59 ± 0.33d |  |
| **P Values** | | T | P<0.001 | | | | | |
|  |  | D (0-28) | P>0.05; 0.075 | | | | | |
|  |  | T x D | P<0.05; 0.007 | | | | | |

*T: treatment, D: day (0, 7, 14, 21, and 28), x: interaction; T-NEms: Thyme nanoemulsion; E-NEms: Eucalyptus nanoemulsion; P-NEms: Peppermint nanoemulsion; 50, 100, 200: Applied doses (ppm); PC-Amitraz: Positive control (amitraz); C-No Dose: Untreated control.

**Table S5**. Mean±SEM values of fallen mites at 1, 3, and 5 days after treatment, computed across all sampling days (0-28 days)

| **n** | **Treatment** | **1 Day** | **3 Day** | **5 Day** | **Overall** |
| --- | --- | --- | --- | --- | --- |
| **5** | **T-NEms50** | 25.92 ± 2.02 | 18.64 ± 1.78 | 1.72 ± 0.15 | 15.43 |
| **5** | **T-NEms100** | 25.68 ± 4.61 | 19.88 ± 3.71 | 2.20 ± 0.25 | 15.92 |
| **5** | **T-NEms200** | 38.24 ± 1.61 | 32.88 ± 1.53 | 2.20 ± 0.21 | 24.44 |
| **5** | **E-NEms50** | 23.72 ± 1.53 | 22.24 ± 4.17 | 1.68 ± 0.08 | 15.88 |
| **5** | **E-NEms100** | 25.40 ± 2.22 | 21.52 ± 2.94 | 1.68 ± 0.25 | 16.20 |
| **5** | **E-NEms200** | 32.64 ± 1.03 | 31.20 ± 1.36 | 1.52 ± 0.26 | 21.79 |
| **5** | **P-NEms50** | 28.56 ± 0.99 | 18.84 ± 1.82 | 1.64 ± 0.12 | 16.35 |
| **5** | **P-NEms100** | 26.40 ± 3.46 | 24.56 ± 3.57 | 1.64 ± 0.20 | 17.53 |
| **5** | **P-NEms200** | 35.76 ± 1.69 | 30.12 ± 0.76 | 1.68 ± 0.22 | 22.52 |
| **5** | **P.C.Amitraz** | 20.56 ± 1.64 | 10.12 ± 0.98 | 1.92 ± 0.36 | 10.87 |
| **5** | **C.No Dose** | 1.60 ± 0.33 | 1.72 ± 0.19 | 1.44 ± 0.28 | 1.59 |
| **P Values** | | T | P < 0.001 | | |
|  |  | Tm (1-5) | P < 0.001 | | |
|  |  | T x Tm | P < 0.001 | | |

*T: treatment, Tm: Time (1, 3, and 5), x: interaction; T-NEms: Thyme nanoemulsion; E-NEms: Eucalyptus nanoemulsion; P-NEms: Peppermint nanoemulsion; 50, 100, 200: Applied doses (ppm); PC-Amitraz: Positive control (amitraz); C-No Dose: Untreated control.
