# Supplementary material for: Colony-Level Efficacy of Mentha piperita, Thymus vulgaris and Eucalyptus globulus Essential Oil Nanoemulsions Against Varroa destructor
Source: Exp Appl Acarol. 2026 May 5;96(4):50. doi: 10.1007/s10493-026-01141-y (PMC13144229; doi:10.1007/s10493-026-01141-y)
Supplement: Supplementary file 3 — Supplementary material 3 (PDF 149.7 kb) [file 10493_2026_1141_MOESM3_ESM.pdf]

Response to the Reviewer 1 Comments on Manuscript

Journal: Experimental and Applied Acarology

Submission ID: f4731363-d7b9-4811-bc4e-88c80db2d2ff

I sincerely thank the reviewer for the thorough, critical, and highly insightful evaluation of my manuscript entitled "*Field Efficacy of Mentha piperita, Thymus vulgaris and Eucalyptus globulus Essential Oil Nanoemulsions Against Varroa destructor*" I truly appreciate the time, expertise, and careful attention devoted to this work.

The reviewer's comments have been extremely valuable in improving the methodological clarity, scientific rigor, and overall robustness of the study. I have carefully revised the manuscript in line with all suggestions, and detailed point-by-point responses are provided below. All changes in the revised manuscript are highlighted in yellow for clarity.

While the revision process has certainly been challenging at times, it has also been highly constructive, and I believe the manuscript has significantly benefited from this detailed review.

Thank you again for your constructive and thought-provoking feedback.

Kind regards,

**Response to Reviewer 1**		
**Line Number**	**Reviewer Comment**	**Author Response**
**Graphical abstract**		
We would also like to add a final note based on our field experience. The application method used in this study, which involves spraying each bee-covered frame, can be relatively labor-intensive, especially in strong colonies. However, we have been applying similar formulations in our apiary for several years and have not observed any negative effects on colony health. On the contrary, based on our observations, colonies treated with these formulations appeared to develop more rapidly during the spring season.<br>In addition, we observed that when the formulation was applied directly to drone brood areas (after uncapping), mites were rapidly affected, suggesting strong contact efficacy under practical conditions. While these observations are not part of the controlled dataset presented here, they have encouraged us to further explore this approach with different essential oils and combinations.<br>We also recognize that high infestation levels can lead to significant colony losses and economic damage in beekeeping operations. The reviewer's comments have been particularly valuable in this regard, and we acknowledge that future studies should focus on evaluating these formulations under more moderate infestation levels.<br>Finally, we plan to extend this work in future studies to investigate not only efficacy but also potential residue levels in honey and beeswax, as well as broader impacts on colony health. Considering that similar nanoemulsion systems are widely used in other fields, including food-related applications, we believe that such approaches may offer practical advantages for beekeeping as well.		
Raw Data	General Commentary	Thank you for your valuable and detailed comments. We appreciate your concerns regarding the validity of the data. In response, the complete raw dataset has now been provided as a CSV file to ensure full transparency and to allow independent verification of the results. We have carefully re-checked all calculations and data entries, including infestation values and SEM calculations, and no computational or data entry errors were identified. We confirm that all values were derived directly from colony-level raw data (n = 5 per group), and SEM values were calculated as SD/√n. We would also like to clarify that the infestation values reported in this study represent the number of mites per 10 g of bees, based on a standardized calculation, and do not

		correspond to percentage infestation. The multiplication factor (×10) was applied only once to normalize mite counts per 10 g of bees, and no additional scaling or conversion to percentage was performed. The manuscript has been revised accordingly to prevent any potential misunderstanding. The relatively high infestation levels observed in this study may be associated with local apiary conditions, seasonal dynamics, and field variability. In addition, the sugar shake method, which is known to underestimate true infestation levels compared to alcohol wash, may also contribute to differences when comparing our results with other studies. This limitation has now been explicitly addressed in the revised manuscript. Regarding colony survival and the relatively high infestation levels, we would like to provide additional contextual clarification. The experimental period followed the honey harvest, during which honey supers were removed, resulting in a higher relative concentration of adult bees per unit sample. In addition, colonies still contained brood, which may have supported ongoing mite reproduction. It is also important to note that no acaricide treatments had been applied for approximately 4–5 months prior to the experiment, which likely contributed to the elevated infestation levels observed at the start of the study. Furthermore, nanoemulsion treatments were applied as sprays directly onto each bee-covered frame, ensuring thorough contact between bees and the formulation and allowing a clear assessment of treatment effects under field conditions. Therefore, the relatively high infestation levels reported here reflect field conditions under limited prior treatment and were intentionally included to represent severe infestation scenarios relevant for evaluating treatment performance under challenging conditions. Despite these high infestation levels, colonies remained alive during the 28-day experimental period, likely due to the relatively short duration of the study and the seasonal timing. This point has also been clarified in the discussion. We hope that the inclusion of the raw dataset and the additional clarifications adequately address the reviewer's concerns.
35	Residue free was not removed from the graphical abstract, as stated in the response to reviewers. This still needs to be removed to avoid overstating the data in this manuscript. Perhaps "no synthetic chemical residues" would get the same point across without being problematic? Also, the smaller arrow pointing downward to the	Thank you for this helpful comment. The graphical abstract has been revised accordingly. The phrase "residue free" has been removed.

	bee still doesn't make sense to me after your response. It feels like bee health should be increasing as efficacy increases, denoted by an upward arrow, as opposed to a downward arrow?	
**Introduction**		
38-126	The authors have sufficiently addressed all concerns and provided a good description of the study's aims.	Thank you for this positive and encouraging comment.
**Materials and Methods**		
242	consider changing "total number of dropped Varroa" to "total number of dislodged Varroa" to limit confusion with mite drop measurements	Thank you for your valuable comment. The term has been revised as suggested to avoid confusion.
267	remove "Significant differences between measurement times were indicated using different letter notations in the tables and figures", as this is not true for all figures, and should be stated in the figure caption rather than in the text	Thank you for your valuable comment. The sentence has been removed.
483-485	Perhaps discuss the longevity of these treatments in colonies. Based on mite drop data, do these provide long-term control, or only immediate knockdown? It can be important here to mention feasibility of repeated applications in beekeeping operations in terms of labor costs for beekeepers.	Thank you for your valuable comment.<br>This sentence has been added.<br>The highest mite reduction observed on the first day after application, followed by lower mite counts on days 3 and 5, indicates that the treatments primarily exert a rapid knockdown effect, with limited persistence over time.
471-475	This is the highlight of the discussion; you do a great job bringing in relevant literature to compare to your study. Please expand a bit more on why the T-NEms200 was most effective. How was this affected by droplet size?	Thank you for your valuable comment.<br>This sentence has been added.<br>This higher efficacy may be attributed to the combined effect of its carvacrol-rich composition and the nanoemulsion formulation, which likely enhanced dispersion and contact with mites. Smaller droplet sizes increase surface area and facilitate more efficient interaction between active compounds and target organisms, thereby improving biological activity (Leong et al., 2009).
514-527	The discussion was greatly improved by the revisions. There are a few limitations of the study that I think should still be discussed: drifting of Varroa between colonies (especially with high mite numbers), using	Thank you for your valuable comment.<br>This sentence has been added.<br>In addition, inter-colony transmission of *Varroa destructor* may have influenced the observed mite reduction levels. Drifting and robbing behaviors are well-documented mechanisms facilitating mite movement between colonies, particularly in apiary settings, and may contribute to

	only 1 apiary location, and poor mite recovery with sugar shakes. Additionally, I think that your response to my comment on practical feasibility and economic viability would be valuable additions to the discussion, as they address the needs of the end-users of this product.	reinfestation or fluctuations in mite populations during field experiments (Peck & Seeley, 2019; Goodwin et al., 2006). Notably, rapid increases in mite levels in neighboring colonies have been associated with robbing of heavily infested or collapsing colonies, which can override spatial separation effects and complicate interpretation of treatment efficacy. Furthermore, mite quantification based on sugar shake methods may underestimate actual infestation levels due to incomplete mite recovery, as this method has been reported to detect fewer mites compared to alcohol wash under certain conditions. Therefore, the efficacy values reported in the present study should be interpreted with caution, as both biological factors (mite drift and reinfestation) and methodological constraints may have influenced the measured mite reduction.
503-514	Additionally, I think that your response to my comment on practical feasibility and economic viability would be valuable additions to the discussion, as they address the needs of the end-users of this product.	Thank you for your valuable comment.<br>This sentence has been added.<br>From a practical perspective, essential oil nanoemulsions offer several advantages, including relatively low production costs and the potential for valorization of agro-industrial by-products. For example, nanoemulsion formulations derived from plant materials such as grapefruit peel have been reported as cost-effective and sustainable alternatives with potential for large-scale production (Çiçek et al., 2024). However, certain limitations should be considered. The application of such formulations may be time-consuming, particularly in strong colonies where achieving uniform distribution can be challenging. In addition, although essential oils are generally regarded as environmentally friendly, repeated applications may be required due to their short-term knockdown effect, which could raise concerns regarding potential residue accumulation and labor costs. Therefore, further studies are needed to optimize application strategies and evaluate long-term feasibility under practical beekeeping conditions.
